# The revised Patient Perception of Patient‐Centeredness Questionnaire: Exploring the factor structure in French‐speaking patients with multimorbidity

**DOI:** 10.1111/hex.13068

**Published:** 2020-04-27

**Authors:** Tu Ngoc Nguyen, Patrice Alain Ngangue, Bridget L. Ryan, Moira Stewart, Judith Belle Brown, Tarek Bouhali, Martin Fortin

**Affiliations:** ^1^ Department of Family Medicine and Emergency Medicine Faculty of Medicine and Health Sciences Université de Sherbrooke Sherbrooke QC Canada; ^2^ Centre for Studies in Family Medicine Department of Family Medicine Schulich School of Medicine & Dentistry Western University London ON Canada; ^3^ Department of Epidemiology and Biostatistics Schulich School of Medicine & Dentistry Western University London ON Canada; ^4^ Centre intégré universitaire de santé et de services sociaux du Saguenay-Lac St-Jean QC Canada

**Keywords:** factor analysis, French‐speaking, multimorbidity, patient‐centred, primary care, questionnaires

## Abstract

**Background:**

The Patient Perception of Patient‐Centeredness (PPPC) questionnaire was revised, and there is a need for the questionnaire to be tested in diverse primary care populations.

**Objectives:**

This study aimed to examine the factor structure of the Revised PPPC questionnaire **(**PPPC‐R) in French‐speaking patients with multimorbidity.

**Design:**

Secondary analysis from baseline data of the French arm of Patient‐Centered Innovations for Persons with Multimorbidity Study (PACEinMM Study).

**Setting and participants:**

Participants were adult patients with multimorbidity attending primary health‐care settings.

**Outcome measures:**

Exploratory factor analyses were applied to examine the factor structure of the PPPC‐R. Cronbach's alpha values were calculated to assess the internal consistency of the whole questionnaire and of each factor explored.

**Results:**

There were 301 participants, mean age 61.0, 53.2% female. The PPPC‐R showed very good internal consistency, with three factors: Patient‐Centered Clinical Method (PCCM) Component 1‐Exploring the health, disease and illness experience + PCCM Component 4‐Enhancing the patient–clinician relationship (Factor 1); PCCM Component 2‐Understanding the whole person (Factor 2); and PCCM Component 3‐Finding common ground (Factor 3). There was a good internal consistency within each factor (Cronbach's *α* = 0.87 for 8 items in Factor 1, 0.77 for 5 items in Factor 2 and 0.87 for 5 items in Factor 3).

**Discussion and conclusions:**

The French PPPC‐R factor structure was in accordance with the underpinning conceptual model and presented with three factors. Further assessment of its validity and reproducibility are needed to allow its use as a measure of patient's perception of patient‐centeredness.

## 1 INTRODUCTION

There have been many innovations to improve quality of care and health‐related outcomes for patients in primary health care in Canada.^1^, ^2^ Patient‐centred care, a model of care that enables patients to be co‐producers or co‐creators of their care, is among these innovations.^3^, ^4^, ^5^ Researchers and policymakers worldwide are working to improve patient‐centred care across health‐care systems.^6^, ^7^, ^8^ Patient‐centred care has been shown to enhance patient satisfaction, patient adherence and improve patient health outcomes.^9^, ^10^, ^11^, ^12^, ^13^ It is essential to understand patients' perceptions and experiences with patient‐centred care so that health‐care providers can tailor and improve the care for these patients. Among the measures available, the Patient Perception of Patient‐Centeredness (PPPC) questionnaire has been widely applied in primary care research in Canada and many other countries.^14^, ^15^, ^16^, ^17^, ^18^, ^19^, ^20^ The original PPPC questionnaire consisted of 14 items and covered 3 interactive components of the Patient‐Centered Clinical Method (PCCM) (exploring health, disease and the illness experience; understanding the whole person; finding common ground).^16^, ^17^, ^18^, ^19^ Recently, the PPPC questionnaire was revised, so that the revised PPPC now contains 18 items (PPPC‐R), intended to map to the current Patient‐Centered Clinical Method which contains four components (the original three and enhancing the patient–clinician relationship).^21^

In Quebec, Canada, the French version of the PPPC questionnaire is currently used but little was known about the measure's psychometric properties. There is a need for the PPPC‐R to be tested in diverse primary care populations. In this study, our focus was patients with multimorbidity who constitute a major proportion of the primary health‐care patients. Multimorbidity, defined as having multiple chronic conditions, has become a primary health‐care concern.^22^ A systematic review on 45 studies conducted from 2007 to 2017 showed that the overall prevalence of multimorbidity was 66.1% (with multimorbidity defined as having ≥2 chronic conditions), and 44.2% (with multimorbidity defined as having ≥3 chronic conditions) in older adults in high‐income countries.^23^ In the United States, the number of Americans with multimorbidity is estimated to be around 81 million by 2020.^24^ In Canada, it was reported that the prevalence of multimorbidity was also significant, from around 30% to 70% in primary care settings and around 17% to 59% in the general population.^25^ The prevalence of multimorbidity was also quite high in French‐speaking communities. In 2016, more than 1.1 million people aged 25 or older in Quebec were having multimorbidity.^26^ According to the French National Surveys, around 25% of the French population over 55 had multimorbidity (2008‐2012).^27^ In a population‐based trial of 5647 participants aged 55 years or older in France (2007‐2009), more than 63% of the participants reported having two or more chronic conditions.^28^ Data from the Belgian Health Interview Surveys (2001‐2008) revealed that one third of the 9482 participants aged 55 years or older had at least two chronic diseases.^29^

Therefore, this study aimed to examine the factor structure of the French revised version of the PPPC, the internal consistency of the whole questionnaire and of its factors in a context of primary care in French‐speaking patients with multimorbidity.

## 2 METHODS

This study was a secondary analysis from the baseline data of the French arm of Patient‐Centered Innovations for Persons with Multimorbidity Study (PACE in MM Study).^30^ The PACE in MM study encompassed two randomized controlled trials, one in Quebec and one in Ontario, Canada, that evaluated complex interventions to improve patient‐centred outcomes for patients with multimorbidity.^30^

In the PACE in MM study, multimorbidity was defined as having ≥3 chronic diseases from a list of 19 self‐reported chronic conditions, including hypertension, hyperlipidaemia, obesity, diabetes, chronic musculoskeletal conditions causing pain or limitation, arthritis and/or rheumatoid arthritis, osteoporosis, stomach problem (reflux, heartburn or gastric ulcer), chronic lung disease (asthma, chronic obstructive pulmonary disease, chronic bronchitis), depression/anxiety, cardiovascular disease (angina, myocardial infarction, atrial fibrillation, poor circulation in the lower limbs), heart failure (including valve problems or replacement), colon problem (irritable bowel, Crohn's disease, ulcerative colitis, diverticulosis), thyroid disorder, any cancer in the previous 5 years (including melanoma, but excluding other skin cancer), kidney disease or failure, stroke/transient ischemic attack, chronic urinary problem and chronic hepatitis.

In this study, only data from participants from Quebec, the French‐speaking province of Canada, were utilized. The recruitment period for this study was from July 2016 to July 2017. Studied participants were adult patients with multimorbidity attending primary health‐care settings. Exclusion criteria included severe cognitive impairment and being unable to read or write. At baseline, all participants were required to provide demographic information such as age, gender, height, weight, socio‐economic status and answer questionnaires about chronic health conditions,^31^ and patient‐centred care as measured by the revised Patient Perception of Patient‐Centered Care (PPPC‐R). The questionnaires were administered by a trained research assistant.

### 2.1 Statistical analysis

Analysis of the data was performed using SPSS for Windows 24.0 (IBM Corp.). Continuous variables are presented as mean ± standard deviation or median (range), and categorical variables as frequencies and percentages.

While the questionnaire is intended to measure patient‐centeredness, there has been no previous work conducted on the factor structure of the revised measure in French‐speaking patients. Therefore, we conducted an exploratory factor analyses to explore the initial factor structure of the PPPC‐R in French‐speaking patients with multimorbidity in primary care in Quebec. More precisely, we carried out a principal components factor analysis of the 18 items. The number of components to be extracted was decided based on eigenvalues greater than 1 (The Scree Plot, Figure S1). As the factors were expected to be correlated, Oblimin rotation was applied. However, we also performed Varimax, which is one of the most widely used rotation methods, for comparison.^32^ The minimum loading for an item to be linked to a factor was 0.30.^33^, ^34^ In case, one item was loaded into two factors, the one with higher loading value will be chosen. We calculated Cronbach's alpha values to assess the internal consistency of each factor explored through the first step. The acceptable value for Cronbach's alpha was recommended to be at least 0.70.^35^ Correlation between factors was assessed with Spearman correlation, and two‐sided *P* values < .05 were considered significant.

## 3 RESULTS

There were 301 participants in this study. The mean age of the participants was 61.0 ± 10.5, 53.2% were female, 65.4% were married, and 22.6% had lower education level. (Table 1).

**TABLE 1:** General characteristics

Variables	N = 301
Age (y)	61.0 ± 10.5
Female	160 (53.2)
Marital status	
Married	197 (65.4)
Single/divorced	89 (29.6)
Widower	15 (5.0)
Education	
Secondary school not completed	68 (22.6)
Secondary school completed	70 (23.3)
Higher education	163 (54.1)
BMI (kg/m^2^)	31.55 ± 6.41
Number of chronic health conditions	5.01 ± 1.82
Prevalence of chronic health conditions	
Hyperlipidaemia	235 (78.1)
Hypertension	209 (69.4)
Obese (BMI ≥ 30)	162 (54.2)
Chronic musculoskeletal conditions causing pain or limitation	154 (51.2)
Diabetes	149 (49.5)
Stomach problem (reflux, heartburn or gastric ulcer)	111 (36.9)
Asthma, COPD or chronic bronchitis	96 (31.9)
Depression/anxiety	91 (30.2)
Cardiovascular disease (angina, myocardial infarction, atrial fibrillation, poor circulation in the lower limbs)	64 (21.3)
Colon problem (irritable bowel, Crohn's disease, ulcerative colitis, diverticulosis)	43 (14.3)
Thyroid disorder	37 (12.3)
Arthritis and/or rheumatoid arthritis	26 (8.6)
Osteoporosis	26 (8.6)
Any cancer in the previous 5 y (including melanoma, but excluding other skin cancer)	22 (7.3)
Kidney disease or failure	20 (6.6)
Stroke/Transient ischemic attack	17 (5.6)
Chronic urinary problem	14 (4.7)
Heart failure (including valve problems or replacement)	11 (3.7)
Chronic hepatitis	1 (0.3)

Note

Continuous data are presented as mean ± standard deviation. Categorical data are shown as n (%).

### 3.1 The PPPC‐R characteristics

Based on the Scree Plot (Figure S1), three factors were extracted. The 18 items were loaded into 3 factors. The factor loading matrix obtained through Oblimin rotation is presented in Table 2, and the results obtained through Varimax rotation are presented in Appendix S1 (Table S1). The number of items loaded into each factor was similar between the two rotation methods, although there were some minor differences in the loading values. Among the 18 questions, cross‐loadings happened in 3 questions (Table 2). All items in this analysis had primary loadings over 0.3.

**TABLE 2:** The factor loading matrix of the PPPC‐R with Oblimin rotation

	Factor loading into each factor			The proportion of variance for each item that can be explained by the factor
	1	2	3	
Factor 1: Exploring health, disease and illness experience + Enhancing relationship				
How satisfied were you with the discussion of your problem?	0.81			0.70
To what extent did you agree with your provider's opinion about the problem?	0.77			0.61
How well do you think the provider understood you on that visit?	0.75			0.63
To what extent was your main problem(s) discussed on that visit?	0.69			0.42
To what extent did the provider explain this problem to you?	0.52			0.48
To what extent does your provider really listen to you?	0.50		0.38	0.64
To what extent do you trust your provider?	0.40			0.54
How much would you say that this provider cares about you as a person?	0.39			0.49
Factor 2: Understanding the whole person				
To what extent does your provider know about your family life?		0.81		0.57
How comfortable are you discussing personal problems related to your health with your provider?		0.67		0.56
To what extent does your provider show you compassion?		0.53		0.61
To what extent does your provider respect your beliefs, values and customs?	0.46	0.52		0.59
To what extent does your provider consider your thoughts and feelings?	0.40	0.50		0.70
Factor 3: Finding common ground				
To what extent did your provider explain treatment?			0.88	0.71
To what extent did the provider explore how manageable this treatment would be for you?			0.85	0.70
To what extent did you and the provider discuss your respective roles?			0.78	0.63
To what extent did the provider ask about your goals for treatment?			0.78	0.65
To what extent did the provider encourage you to take the role you wanted in your own care?			0.59	0.67

Note

Extraction Method: Principal Component Analysis. Rotation Method: Oblimin with Kaiser Normalization. These 3 factors explained 60.5% of the variance (Factor 1:46.2%, Factor 2:6.1% and Factor 3:8.3%).

The gray differenciates among the factors.

Upon examination, the items within each of these three factors corresponded to the four components of the PCCM as follows: Factor 1–PCCM Component 1 (Exploring health, disease and illness experience) + PCCM Component 4 (Enhancing patient–clinician relationship), Factor 2–PCCM Component 2 (Understanding the whole person) and Factor 3–PCCM Component 3 (Finding common ground).

There was a good internal consistency within each factor (Cronbach's *α* = 0.87 for 8 items in Factor 1, Cronbach's *α* = 0.77 for 5 items in Factor 2 and Cronbach's *α* = 0.87 for 5 items in Factor 3) (Table 3).

**TABLE 3:** Internal consistency and inter‐correlations of the three factors of the PPPC‐R

Factors	Cronbach's *α*	Factor 1	Factor 2
Factor 1. Exploring health, disease and illness experience + Enhancing relationship	0.87		
Factor 2. Understanding the whole person	0.77	*r* = .60*	
Factor 3. Finding common ground	0.87	*r* = .60*	*r* = .45*

* *P* < .001. *r*: correlation coefficient.

A description of the French version of the PPPC‐R questionnaire is presented in Table S2.

## 4 DISCUSSION

In this study of 301 participants with multimorbidity, the French version of the PPPC‐R showed three distinct factors with good internal consistency. The consistency value found in this study was similar to previous studies using the previous 14‐item version of this questionnaire in evaluating patient's perception of patient‐centred care.^18^, ^19^, ^36^

In this study in French‐speaking patients with multimorbidity, exploratory factor analysis identified three factors describing patient‐centred care: (a) Exploring health, disease and illness experience + Enhancing patient–clinician relationship, (b) Understanding the whole person and (c) Finding common ground. This finding is compatible with the four components of the Patient‐Centered Clinical Method. However, there was a fusion of two components of the model into Factor 1: ‘Exploring health, disease and illness experience’ and ‘Enhancing patient–clinician relationship’. It was an overlap that can be expected as a question may cover two different components of the PCCM model at the same time. In a previous systematic review on the measurement of patient perception of patient‐centred care, the PPPC questionnaire with 14 items was described as lacking an assessment of patient–clinician relationship.^37^ The PPPC‐R represents in this respect an improvement.

The findings of this study contribute to the evidence gap in the application of patient‐centred care measures. The PPPC questionnaire has been used in studies of different populations such as breast cancer patients,^16^, ^19^ older patients^20^ and patients in family practice,^15^, ^18^ but no study has reported its use in patients with multimorbidity more specifically. Although the instrument was previously used in a French population,^38^ this is the first study to explore the applicability of this questionnaire in patients with multimorbidity in primary care, using the French version of the PPPC questionnaire in its most recent revised version. However, given this was a first assessment in French, further work is needed to confirm the factorial structure.

This study has limitations. We used the whole sample under study for this analysis. No other measure was available for assessing validity. As the questionnaires were administered by an interviewer, it may have amplified a desirability bias. It is unclear how it could have affected the results.

## 5 CONCLUSION

In this study in French‐speaking patients with multimorbidity, the PPPC‐R factor structure was in accordance with the underpinning conceptual model and presented with three factors. Further assessment of its validity, reproducibility and responsiveness is needed to allow its use as a measure of patient's perception of patient‐centeredness in research and clinical care for patients with multimorbidity.

## CONFLICT OF INTEREST

All authors have no conflict of interests to declare.

## ETHICAL APPROVAL

The PACE in MM study was approved by the Comité d’éthique de la recherche du Centre intégré universitaire de santé et de services sociaux du Saguenay–Lac‐St‐Jean.

## Supporting information

Appendix S1

## Data Availability

The data that support the findings of this study are available upon request from the corresponding author. The data are not publicly available due to privacy or ethical restrictions.
