# Supplementary material for: The revised Patient Perception of Patient‐Centeredness Questionnaire: Exploring the factor structure in French‐speaking patients with multimorbidity
Source: Health Expect. 2020 Apr 27;23(4):904–9. doi: 10.1111/hex.13068 (PMC7495072; doi:10.1111/hex.13068)
Supplement: Supplementary file 1 — Appendix S1 [file HEX-23-904-s001.docx]

**Appendix**

**Supplementary Table 1. The factor loading matrix of the PPPC-R with Varimax rotation**

|  | Component | | |
| --- | --- | --- | --- |
|  | 1 | 2 | 3 |
| **Factor 1: Exploring health, disease and illness experience + Enhancing relationship** | | | |
| How satisfied were you with the discussion of your problem? | 0.78 |  |  |
| To what extent did you agree with your provider’s opinion about the problem? | 0.73 |  |  |
| How well do you think the provider understood you on that visit? | 0.73 |  |  |
| To what extent was your main problem(s) discussed on that visit? | 0.63 |  |  |
| To what extent does your provider really listen to you? | 0.56 | 0.51 |  |
| To what extent did the provider explain this problem to you? | 0.56 |  | 0.33 |
| To what extent do you trust your provider? | 0.49 | 0.41 | 0.36 |
| How much would you say that this provider cares about you as a person? | 0.48 | 0.32 | 0.39 |
| **Factor 2: Understanding the whole person** |  |  |  |
| To what extent does your provider know about your family life? |  | 0.75 |  |
| How comfortable are you discussing personal problems related to your health with your provider? |  | 0.69 |  |
| To what extent does your provider show you compassion? | 0.40 | 0.61 |  |
| To what extent does your provider consider your thoughts and feelings? | 0.50 | 0.61 |  |
| To what extent does your provider respect your beliefs, values and customs? | 0.49 | 0.59 |  |
| **Factor 3: Finding common ground** |  |  |  |
| To what extent did your provider explain treatment? |  |  | 0.81 |
| To what extent did the provider explore how manageable this treatment would be for you? |  |  | 0.80 |
| To what extent did you and the provider discuss your respective roles? |  |  | 0.74 |
| To what extent did the provider ask about your goals for treatment? |  |  | 0.74 |
| To what extent did the provider encourage you to take the role you wanted in your own care? | 0.36 | 0.39 | 0.63 |
| Extraction Method: Principal Component Analysis.  Rotation Method: Varimax with Kaiser Normalization. | | | |

**Supplementary Table 2. The French version of the revised PPPC questionnaire**

About your last visit with your healthcare provider.

À propos de votre dernière visite avec votre professionnel de santé.

| **Factor** **1: Exploring health, disease and illness experience + Enhancing relationship** |
| --- |
| **How satisfied were you with the discussion of your problem?**  1: very satisfied, 2: satisfied, 3: somewhat satisfied, 4: not satisfied  *À quel point êtes-vous satisfait de la discussion concernant votre problème?*  *1: Très satisfait, 2: satisfait, 3: quelque peu satisfait, 4: insatisfait* |
| **To what extent did you agree with your provider’s opinion about the problem?**  1: completely, 2: mostly, 3: a little, 4: not at all  *Dans quelle mesure étiez-vous d’accord avec l’opinion du professionnel de la santé concernant votre problème?*  *1: Complètement, 2: en bonne partie, 3: un peu, 4: pas du tout* |
| **How well do you think the provider understood you on that visit?**  1: very well, 2: well, 3: somewhat, 4: not at all  *À quel point pensez-vous que le professionnel de la santé vous a compris lors de cette visite?*  *1: Très bien, 2: bien, 3: quelque peu, 4: pas du tout* |
| **To what extent was your main problem(s) discussed on that visit?**  1: completely, 2: mostly, 3: a little, 4: not at all  *Dans quelle mesure a-t-il été discuté de votre (vos) principal(aux) problème(s) de santé lors de cette visite?*  *1: Complètement, 2: En bonne partie, 3: un peu, 4: Pas du tout* |
| **To what extent did the provider explain this problem to you?**  1: completely, 2: mostly, 3: a little, 4: not at all  *Dans quelle mesure le professionnel de la santé vous a-t-il expliqué la nature de votre problème?*  *1: Complètement, 2: en bonne partie, 3: un peu, 4: pas du tout* |
| **To what extent does your provider really listen to you?**  1: completely, 2: mostly, 3: a little, 4: not at all  *Dans quelle mesure votre professionnel de la santé vous écoute-t-il réellement?*  *1: Complètement, 2: en bonne partie, 3: un peu, 4: pas du tout* |
| **To what extent do you trust your provider?**  1: completely, 2: mostly, 3: a little, 4: not at all  *Dans quelle mesure pouvez-vous compter sur votre professionnel de la santé?*  *1: Complètement, 2: en bonne partie, 3: un peu, 4: pas du tout* |
| **How much would you say that this provider cares about you as a person?**  1: Very much, 2: a fair amount, 3: a little, 4: not at all  *Dans quelle mesure diriez-vous que ce professionnel de la santé prend soin de vous en tant que personne?*  *1: beaucoup, 2: assez, 3: un peu, 4: pas du tout* |
| **Factor** **2: Understanding the whole person** |
| **To what extent does your provider know about your family life?**  1: completely, 2: mostly, 3: a little, 4: not at all  *Dans quelle mesure votre professionnel de la santé connaît-il votre vie familiale?*  *1: Complètement, 2: en bonne partie, 3: un peu, 4: pas du tout* |
| **How comfortable are you discussing personal problems related to your health with your provider?**  1: completely, 2: mostly, 3: a little, 4: not at all  *Dans quelle mesure vous sentez-vous à l’aise à discuter de problèmes d’ordre personnel liés à votre santé avec votre professionnel de la santé?*  *1: Complètement, 2: en bonne partie, 3: un peu, 4: pas du tout* |
| **To what extent does your provider show you compassion?**  1: completely, 2: mostly, 3: a little, 4: not at all  *Dans quelle mesure votre professionnel de la santé vous démontre-t-il de la compassion?*  *1: Complètement, 2: en bonne partie, 3: un peu, 4: pas du tout* |
| **To what extent does your provider respect your beliefs, values and customs?**  1: completely, 2: mostly, 3: a little, 4: not at all  *Dans quelle mesure votre professionnel de la santé respecte-t-il vos croyances, vos valeurs et vos coutumes?*  *1: Complètement, 2: en bonne partie, 3: un peu, 4: pas du tout* |
| **To what extent does your provider consider your thoughts and feelings?**  1: completely, 2: mostly, 3: a little, 4: not at all  *Dans quelle mesure votre professionnel de la santé tient-il compte de vos opinions et vos sentiments?*  *1: Complètement, 2: en bonne partie, 3: un peu, 4: pas du tout* |
| **Factor** 3**: Finding common ground** |
| **To what extent did your provider explain treatment?**  1: very well, 2: well, 3: somewhat, 4: not at all  *Dans quelle mesure votre professionnel de la santé vous a-t-il expliqué le traitement?*  *1: Très bien, 2: bien, 3: quelque peu, 4: pas du tout* |
| **To what extent did the provider explore how manageable this treatment would be for you?**  1: completely, 2: mostly, 3: a little, 4: not at all  *Dans quelle mesure le professionnel de la santé a-t-il exploré ce que sera la gestion (du traitement) pour vous?*  *1: Complètement, 2: en bonne partie, 3: un peu, 4: pas du tout* |
| **To what extent did you and the provider discuss your respective roles?**  1: completely, 2: mostly, 3: a little, 4: not at all  *Dans quelle mesure avez-vous discuté avec votre professionnel de la santé de vos rôles respectifs?*  *1: Complètement, 2: en bonne partie, 3: un peu, 4: pas du tout* |
| **To what extent did the provider ask about your goals for treatment?**  1: completely, 2: mostly, 3: a little, 4: not at all  *Dans quelle mesure le professionnel de la santé vous a-t-il questionné sur vos buts concernant le traitement?*  *1: Complètement, 2: en bonne partie, 3: un peu, 4: pas du tout* |
| **To what extent did the provider encourage you to take the role you wanted in your own care?**  1: completely, 2: mostly, 3: a little, 4: not at all  *Dans quelle mesure le professionnel de la santé vous a-t-il encouragé à prendre le rôle que vous désiriez pour vos soins?*  *1: Complètement, 2: en bonne partie, 3: un peu, 4: pas du tout* |


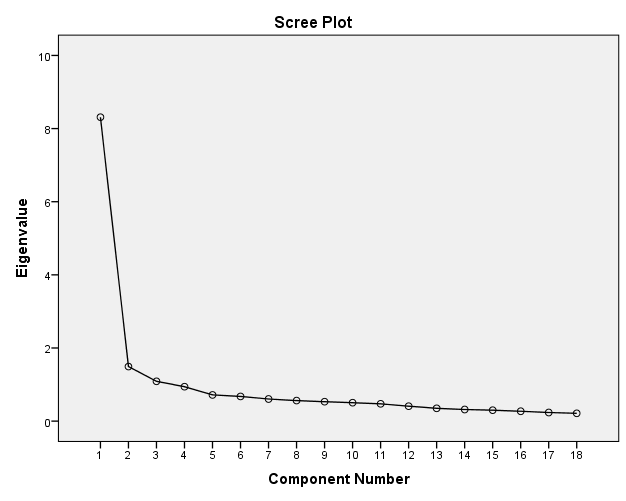


**Supplementary Figure 1. The Scree plot of the eigenvalues**
